# Contour recognition of complex leaf shapes

**DOI:** 10.1371/journal.pone.0189427

**Published:** 2017-12-08

**Authors:** Giacomo Diaz

**Affiliations:** Department of Biomedical Sciences, University of Cagliari, Cagliari, Italy; Universita degli Studi di Trento, ITALY

## Abstract

The leaf shape is an important taxonomic character. Compared to the classic morphological leaf features such as veins, margin indentations, sinuses, etc., the shape is simpler to obtain by using the 'magic wand' or other contouring tools that are available in most of imaging applications. The only exception is when leaves develop large lobes that get in touch or overlap each other, as the presence of hidden or closed portions of the leaf border precludes the application of automatic methods and forces the leaf contour to be traced manually. This is a time consuming and relatively accurate operation that, nevertheless, can not be avoided, as overlapping lobes are characteristic features of the leaves of several plant species and varieties. The method described in the paper overcomes this problem as it allows the leaf contour to be achieved even in the presence of touching or overlapping lobes. The method involves three steps: (1) the acquisition of leaf images using a transilluminator, (2) a two-level image segmentation that allows all leaf components (blade, overlapping lobes and closed sinuses) to be represented in a single binary image, and (3) the contouring and concatenation of all binary outlines in a single, self-intersecting closed contour that reproduces accurately the leaf shape. The method can be extended to acquire the shape of leaves of herbarium specimens, that are often overlapped but can not be easily handled and repositioned because of their extreme fragility and relevant taxonomic value.

## Introduction

**Fig 1:**
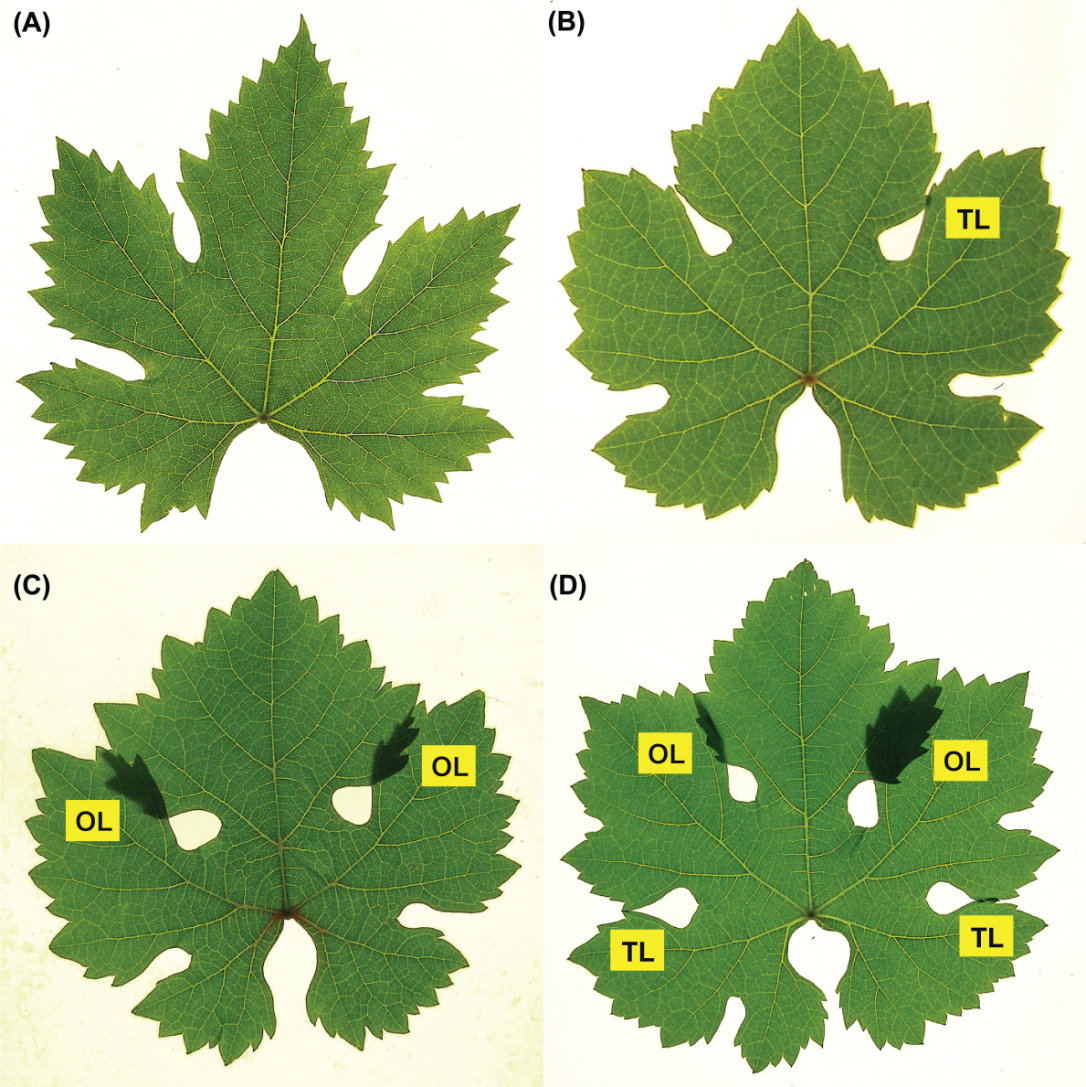
Examples of grapevine leaf shapes. (A) Simple leaf. (B) Leaf with touching lobes [TL]. (C) Leaf with overlapping lobes [OL]. (D) Leaf with both touching and overlapping lobes [TL, OL]. Automatic threshold and contouring methods can be applied only to (A).

The leaf shape is an important character of plant taxa. Leaf shapes may be evaluated by numerical descriptors of different complexity such as aspect ratio, circularity, solidity, fractal dimension, harmonic components, etc. [1–6]. However, all shape descriptors require the preliminary acquisition of the leaf contour. This is generally achieved by automatic contouring tools, popularly known as 'magic wand', that are available in most of imaging applications. On the other hand, in some plants, typically in grapevine (Vitis vinifera), but also in maple (Acer nigrum), figs (Ficus carica), chrysanthemun and other species, leaves develop large lobes that expand laterally up to touch and overlap each other. In this case a portion of the leaf contour is closed or hidden by touching lobes (TL) and overlapping lobes (OL) (Fig 1) and, to my knowledge, no methods are still available to capture such complex leaf shapes. The only alternative is manual tracing, an operation that is time consuming and does not ensure the accuracy of automatic methods, but, nevertheless, can not be omitted, as TL and OL are important taxonomic characters not only at the level of species but also at that of subspecies and varieties. This has been experimentally confirmed by in a study which analyzed the leaf images of 12 different varieties of chrisanthemum by the curvature scale space method, considering or not considering the intersections of the leaf outline produced by OL [7]. Data showed that classification of chrisanthemum varieties was significantlt improved by including OL in the leaf outline.

**Fig 2:**
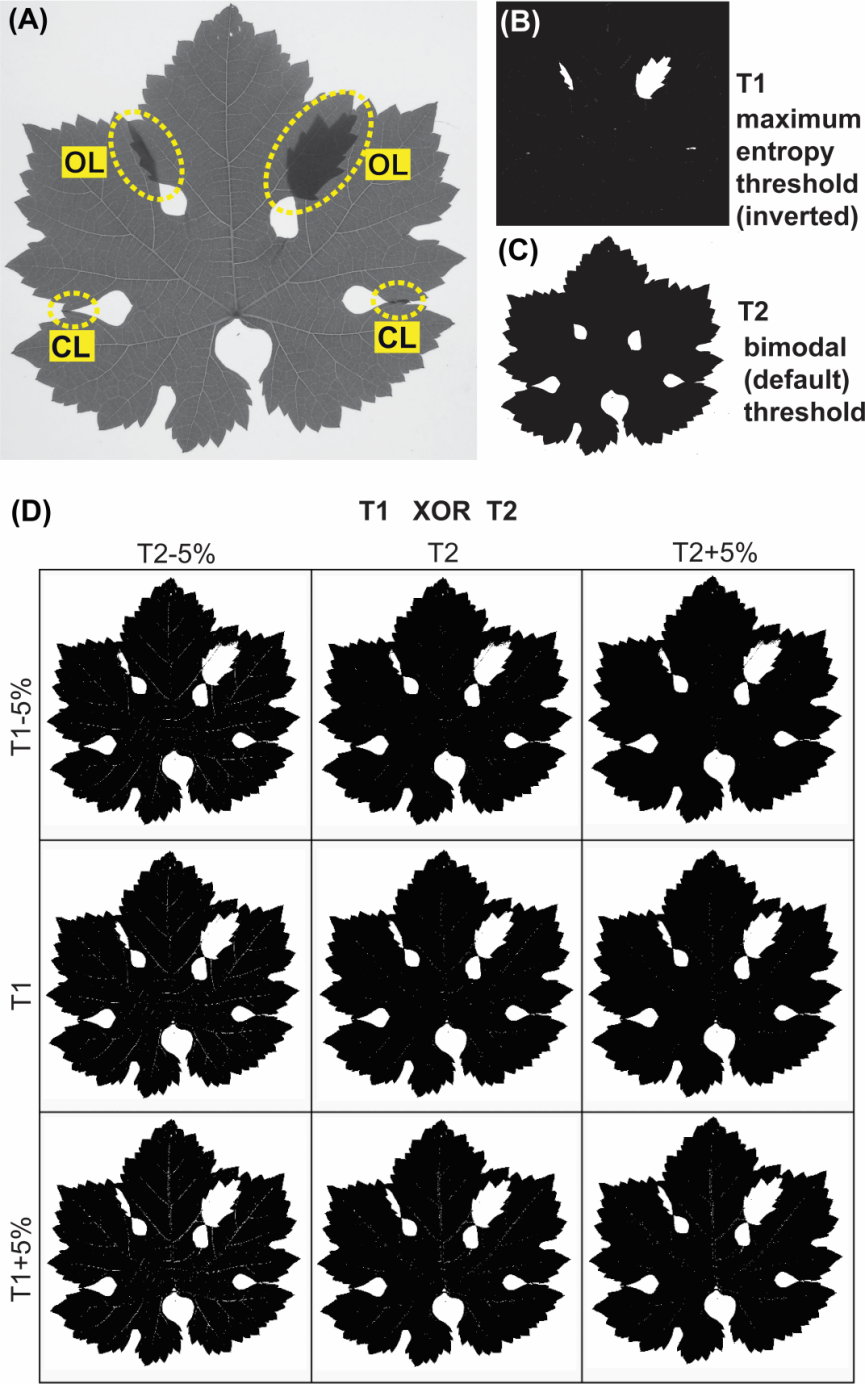
Two-level segmentation of a grapevine leaf with overlapping lobes (OL) and touching lobes (TL). (A) Original image obtained with a transilluminator. (B) Negative of the binay mask [T1] obtained with the maximum entropy threshold method, showing OL as small white outline. (C) Binary mask obtained with the default (bimodal) threshold method [T2], showing the whole leaf outline as a black outline. (D) Panel of nine XORed images obtained by combining slightly changed T1 and T2 threshold values (± 5% T1 and ±5% T2, respectively), showing OL as white outlines within the black outline of the leaf blade. Leaf sinuses closed by TL and OL can also be detected.

A method for the acquisition of leaf contours with TL and OL, implemented in a ImageJ/Fiji macro, is described in this paper. The procedure consists in the acquisition of leaf images in transillumination and in the next segmentation of all leaf components (blade, OL, TL and sinuses) to be represented in a single binary image (Fig 2). Binary outlines are then separately contoured and concatenated in a single, self-intersecting closed contour that reproduces accurately the leaf shape. The method can also be employed to acquire the contour of leaves of herbarium specimens that are often overlapped but, because of their considerable fragility and taxonomic value, can not be handled or repositioned. A method for the automatic extraction of leaf characters from herbarium specimens has been already proposed [8] but using standard (i.e., frontally illuminated rather than transilluminated) images that prevent the detection of overlaps.

## Methods and results

### Image acquisition

Leaf images are acquired using a transilluminator or a scanner for transparencies with photographic resolution (300 dpi and gray levels) and saved in a loss-less image format (i.e., TIF, BMP or PNG). Lossy formats, such as JPG, are unsuitable. Leaves should be adequately pressed on the scan bed to ensure that all parts result in-focus. In addition, leaves should be not damaged and, in general, fully developed. However, the criterion of leaf selection may vary consistently with the aim of the study (taxonomic, developmental, evolutionary, genome/transcriptome-related, etc. [2]). The number of leaves depends on the level of accuracy and margin of error needed, which in turn depends on the natural variability of the leaf shape, that differs from species to species.

### Multilevel segmentation

In transilluminated images, OL result as small regions of higher density compared to the rest of the leaf blade (Figs 1C,1D and 2A). These regions can be selectively segmented by threshold methods based on maximum entropy [9] (Fig 2B). Small traces due to thick venations can be removed by a binary 'close' operation. Conversely, the whole leaf blade can be segmented using automatic (default) threshold methods that assume a bimodal histogram (Fig 2C). If we define T1 the inverted binary mask obtained by the maximum entropy threshold, and T2 the binary mask obtained by the default threshold, then a XOR operation between the T1 and T2 results in a third mask where OL, closed sinuses and the background appear as distinct white areas, and the rest of the leaf blade as a black area. However, in the light of the fact that all automated threshold methods are affected by the image composition (i.e., the ratio between leaf and background areas) and illumination (i.e., non linear brightness and contrast settings of the scanner), the obained T1 and T2 masks may not be optimal. Instead it might be useful to have a panel of several XORed images with slightly varied T1 and T2 values. For example, as Fig 2D shows, nine images obtained with T1±5% and T2±5%, to choose the most suitable combination. All these operations do not concern leaves exhibiting only TL, as these leaves require only a single default (bimodal) threshold.

### Multiple contour acquisition and concatenation

**Fig 3:**
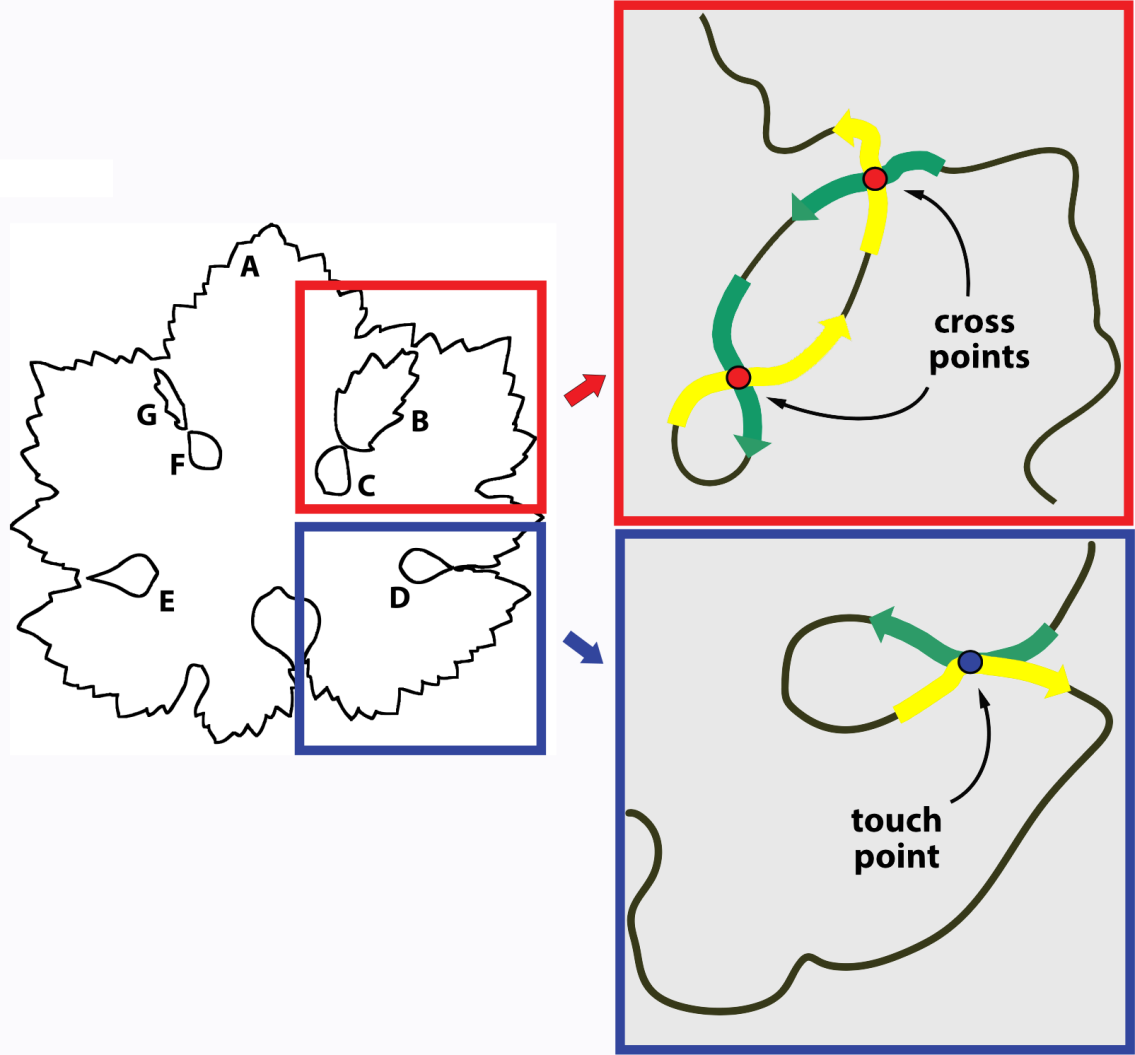
Identification of overlapping and touch points. The image shows the seven contours (labeled A to G) obtained from a composite mask of Fig 1D. [A] is the main leaf blade; [B] and [G] are OL; [C] and [F] are leaf sinuses closed by OL; [D] and [E] are leaf sinuses closed by TL. In the enlarged red frame (simplified), the green and yellow arrows show the path of the self-intersecting or slalom curve which concatenates [A], [B] and [C] contours passing through two cross points (red dots). In the enlarged blue frame, the green and yellow arrows show the path of the self-contacting curve which concatenates the [D] and [A] contours, passing through the touch point (blue dot).

**Fig 4:**
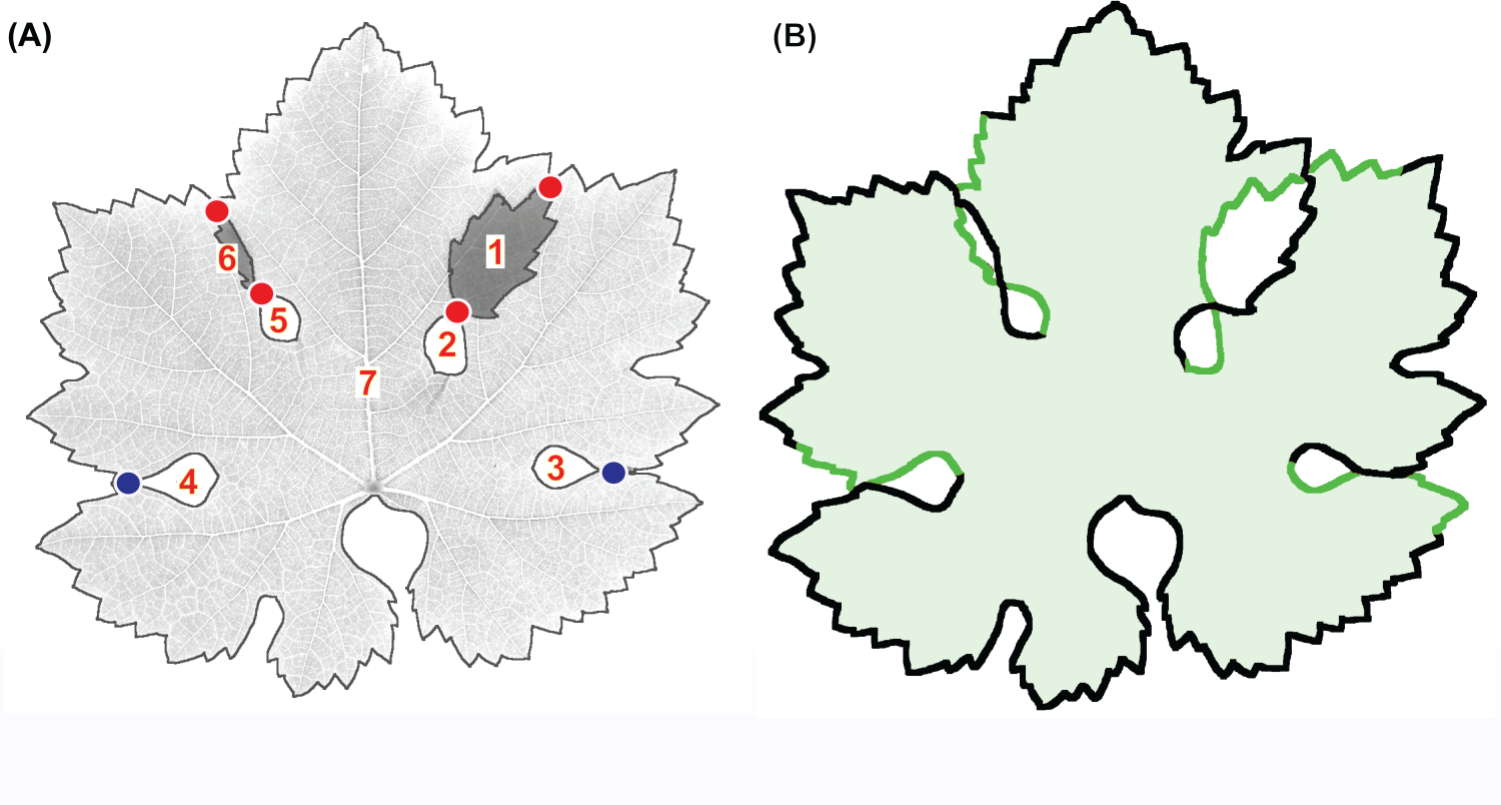
Concatenation of TL and OL contours. (A) Localization of cross points (red dots) and touch points (blue dots) between the seven contours of Fig 3. (B) Result of contour concatenation. The correct link of the contours is highlighted by the sequence of black and green traces.

**Fig 5:**
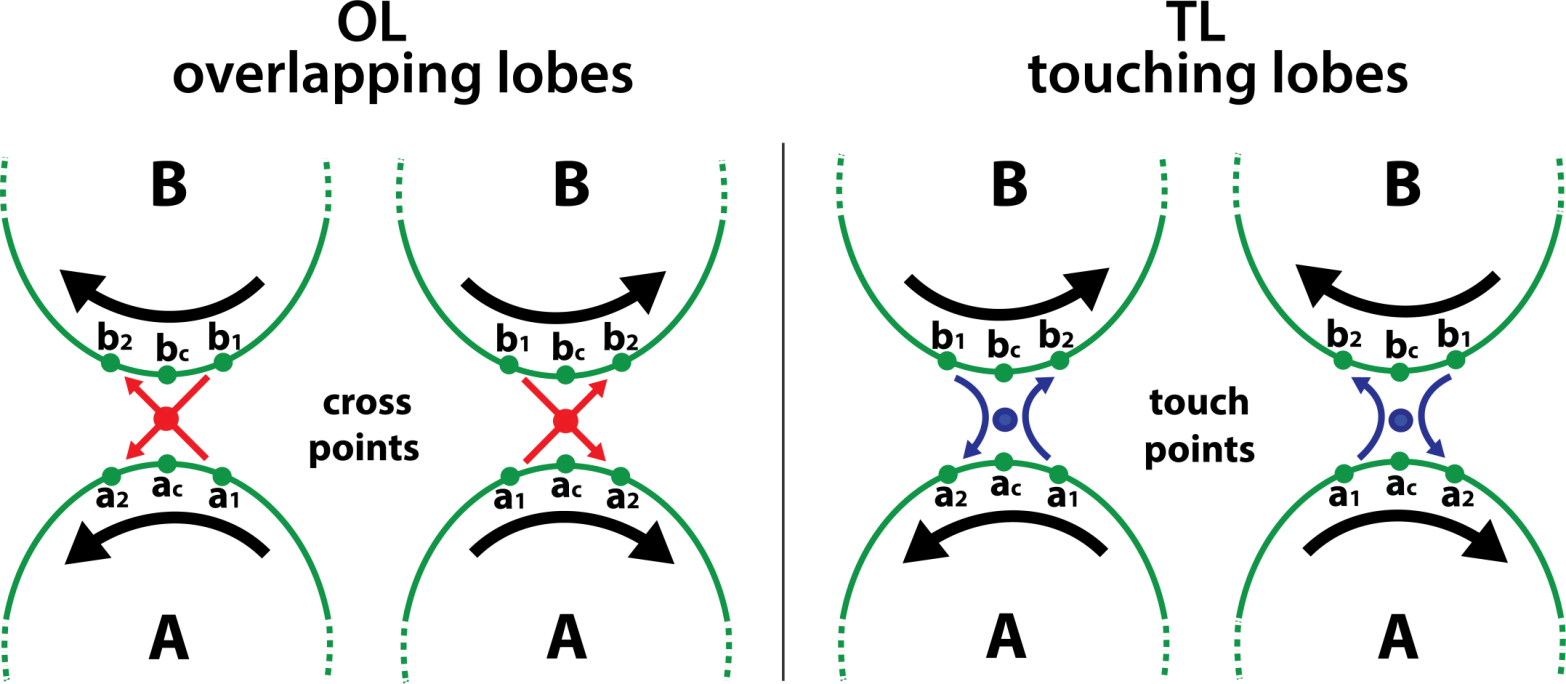
Concatenation algorithm. The diagram shows the coordinate orientations (black arrows) needed to join adjacent contours (**A** and **B**) produced by OL (left panel) or TL (right panel) binary outlines. The red and blue dots indicate the cross points and touch points, respectively. The correct contour orientation and concatenation are achieved through the following steps.

The black and white outlines of the selected mask can be easily contoured using the classic 'magic wand' tool (Fig 3). The contours are automatically saved as coordinate arrays. However, it must be noted that contour coordinates may be clockwise or counter-clockwise oriented, depending on the location of the outline pointed by the 'magic wand'. This point is of fundamental importance, as different orientations and linking strategies are required to join the contours of TL and OL outlines. In particular, OL outlines show a contour that self-intersects two times, following a sort of *slalom* through a pair of cross points (Fig 3, red frame). Conversely, TL outlines show a contour that does not intersect, but self-contacts in correspondence of a touch point (Fig 3, blue frame). Thus, TL and OL must be first identified and then the cross and touch points are localized on the image (Fig 4A). The next concatenation of contours (Fig 4B) is somewhat complex but is fully automatic. The algorithm is described in Fig 5.

First, we identify, in **A** and **B**:

- the points **a**_**c**_ and **b**_**c**_ that are closest to the cross or touch point
- the points **a**_**1**_ and **b**_**1**_ that precede **a**_**c**_ and **b**_**c**_, in the contour orientation (index 1 = index c-1)
- the points **a**_**2**_ and **b**_**2**_ that follow **a**_**c**_ and **b**_**c**_, in the contour orientation (index 2 = index c+1)

Let **p** be the line segment connecting the points **a**_**1**_ and **b**_**2,**_ and **q** the line segment connecting the points **a**_**2**_ and **b**_**1.**_ Then, we test whether **p** and **q** intersect or not.

The regression parameters of **p** and **q** segments are:

$$p_{slope}=\frac{\left(a_{1y}-b_{2y}\right)}{\left(a_{1x}-b_{2x}\right)}$$

$$p_{intercept}=\frac{a_{1y}+b_{2y}}{2}-p_{slope}\times \frac{a_{1x}+b_{2x}}{2}$$

$$q_{slope}=\frac{\left(a_{2y}-b_{1y}\right)}{\left(a_{2x}-b_{1x}\right)}$$

$$q_{intercept}=\frac{a_{2y}+b_{1y}}{2}-q_{slope}\times \frac{a_{2x}+b_{1x}}{2}$$

The common range of **p** and **q** coordinates is delimited by:

$$R_{xmin}=minOf(a_{1x},b_{2x},a_{2x},b_{1x})$$

$$R_{xmax}=maxOf(a_{1x},b_{2x},a_{2x},b_{1x})$$

$$R_{ymin}=minOf(a_{1y},b_{2y},a_{2y},b_{1y})$$

$$R_{ymax}=maxOf(a_{1y},b_{2y},a_{2y},b_{1y})$$

Except that in the case of parallelism, the lines to which **p** and **q** segments belong intersect at the point **Z** with coordinates:

$$Z_{x}=\frac{\left(p_{intercept}-q_{intercept}\right)}{\left(q_{slope}-p_{slope}\right)}$$

$$Z_{y}=p_{intercept}+p_{slope}\times Z_{x}$$

But **p** and **q** segments intersect if and only if the **Z** coordinates fall within the common range of **p** and **q** coordinates, that is:

$$Z_{x}\geq R_{xmin}\;AND\;Z_{x}\leq R_{xmax}\;AND\;Z_{y}\geq R_{ymin}\;AND\;Z_{y}\leq R_{ymax}$$

At this point, the **A** and **B** contours produced by OL can be concatenated only if **p** and **q** segments intersect (red arrows). If **p** and **q** do not intersect, the orientation of one of the two contours must be inverted. Conversely, the **A** and **B** contours produced by TL can be concatenated if and only if **p** and **q** do not intersect (blue arrows). If **p** and **q** segments intersect, the orientation of one of the two contours must be inverted. In both cases, concatenation is obtained through the following steps:

- contour **A** opens in **a**_**c**_
- **a**_**c**_ links to **b**_**c**_
- the coordinate chain continues making a complete tour of contour **B**, following its orientation, up to reach **b**_**c**_ again
- **b**_**c**_ links again to **a**_**c**_
- the coordinate chain continues making a complete tour of contour **A**, following its orientation, up to reach **a**_**c**_ again

This results in a new, closed contour that replaces **A** and **B** contours. After each concatenation, the number of contours is reduced by 1, so that the algorithm is repeated untill all contours are eventually joined to produce a single, closed leaf contour.

**Fig 6:**
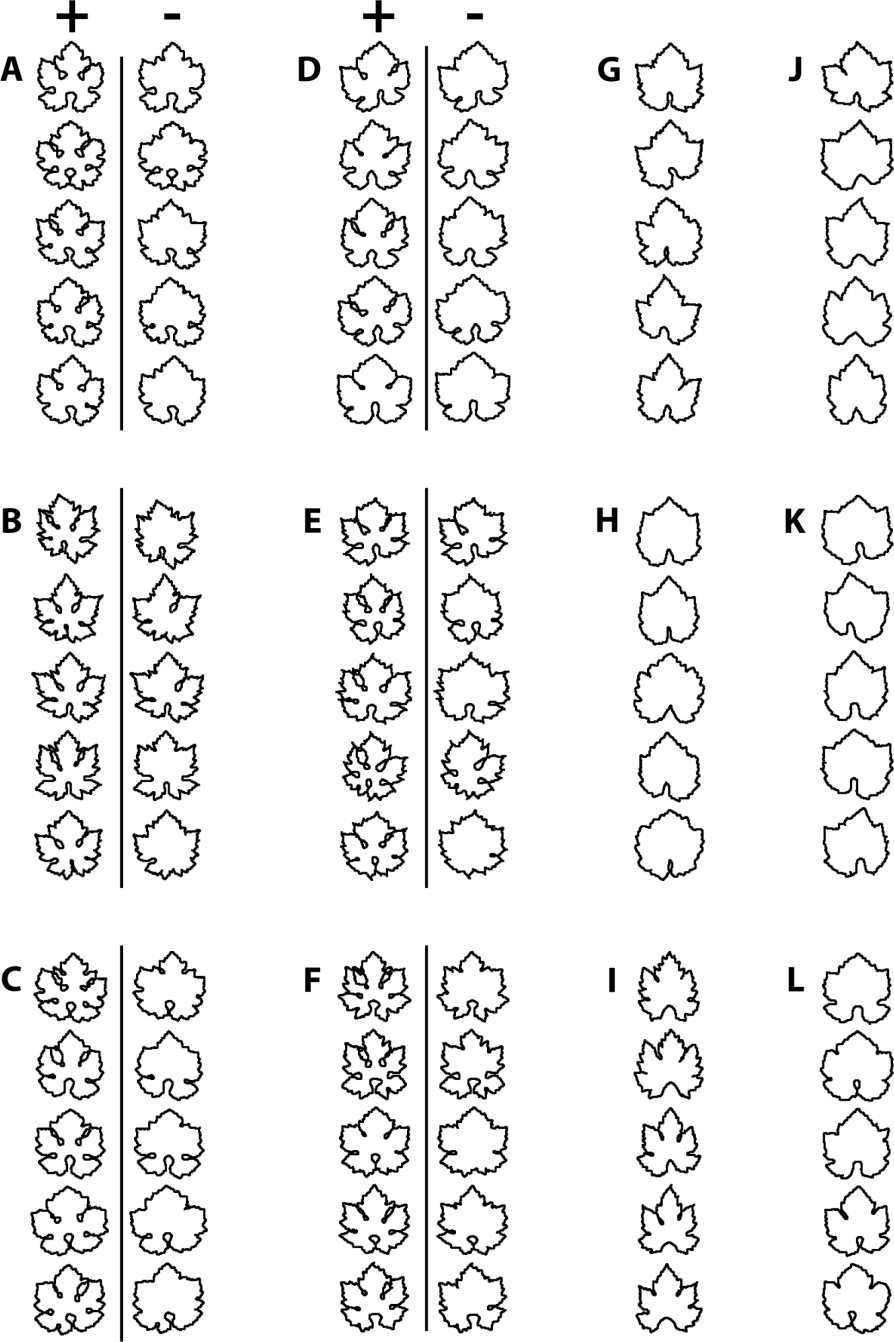
Visual comparison of leaf shapes obtained by considering or not considering TL or OL. Leaves of six grapevine cultivars exhibiting TL and OL [A-F] were achieved considering (+ columns) or not considering (- columns) TL and OL. To do this, the same images were processed two times: first using the dual threshold and contouring method illustrated in this paper, and then using the default threshold method. Other cultivars that did not exhibit TL or OL [G-L] were processed using the default threshold method.

**Fig 7:**
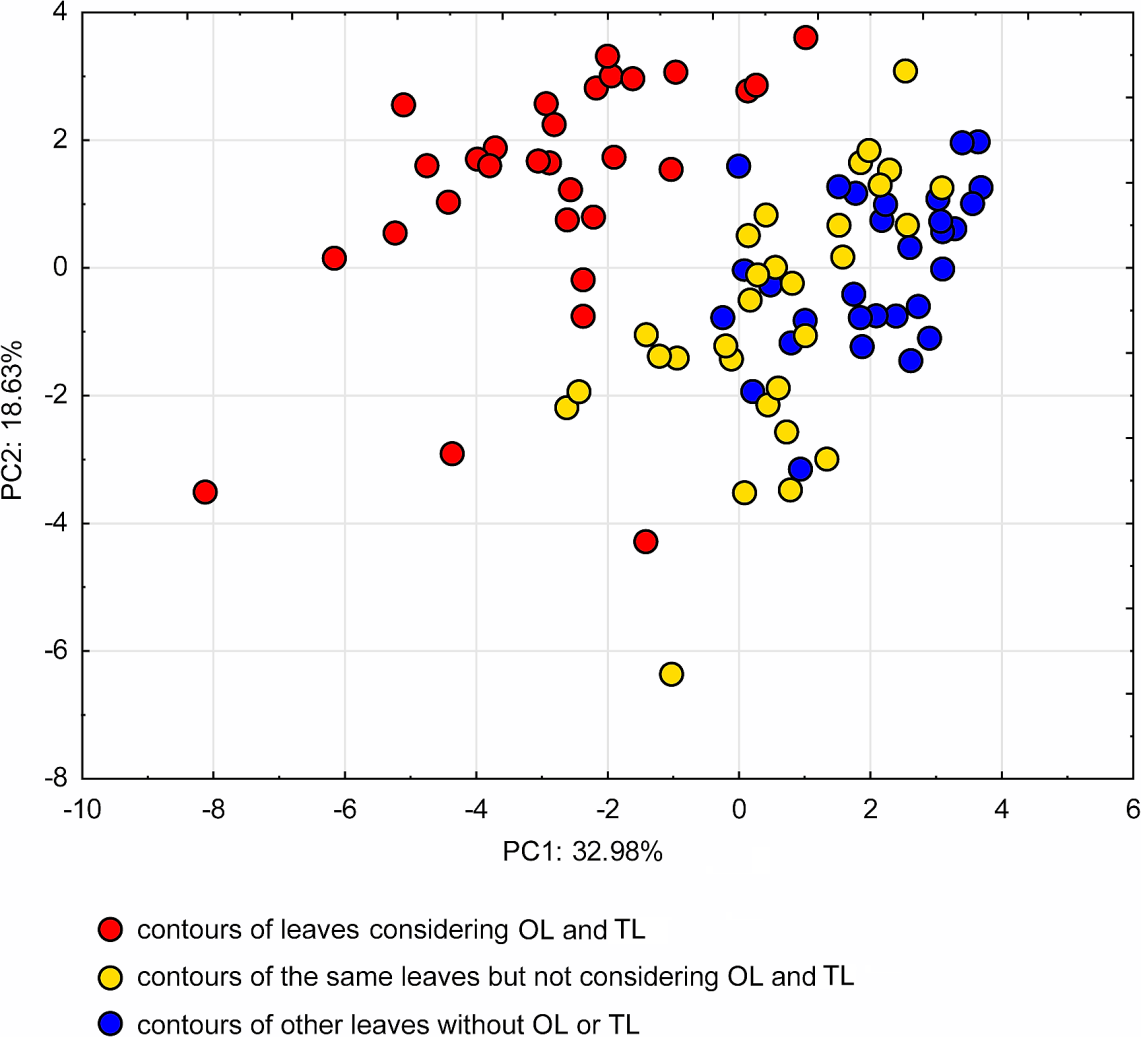
Statistical comparison of leaf shapes obtained by considering or not considering TL or OL. Principal component analysis of the first 20 EFA descriptors (elliptic harmonics) of the 90 leaf contours shown in Fig 6. The plot shows a sharp separation of the leaf shapes of A-F cultivars achieved considering (red dots) or not considering TL and OL (yellow dots). The latter are mixed with the leaves of cultivars G-L, that did not exhibit TL or OL (blue dots).

**Fig 8:**
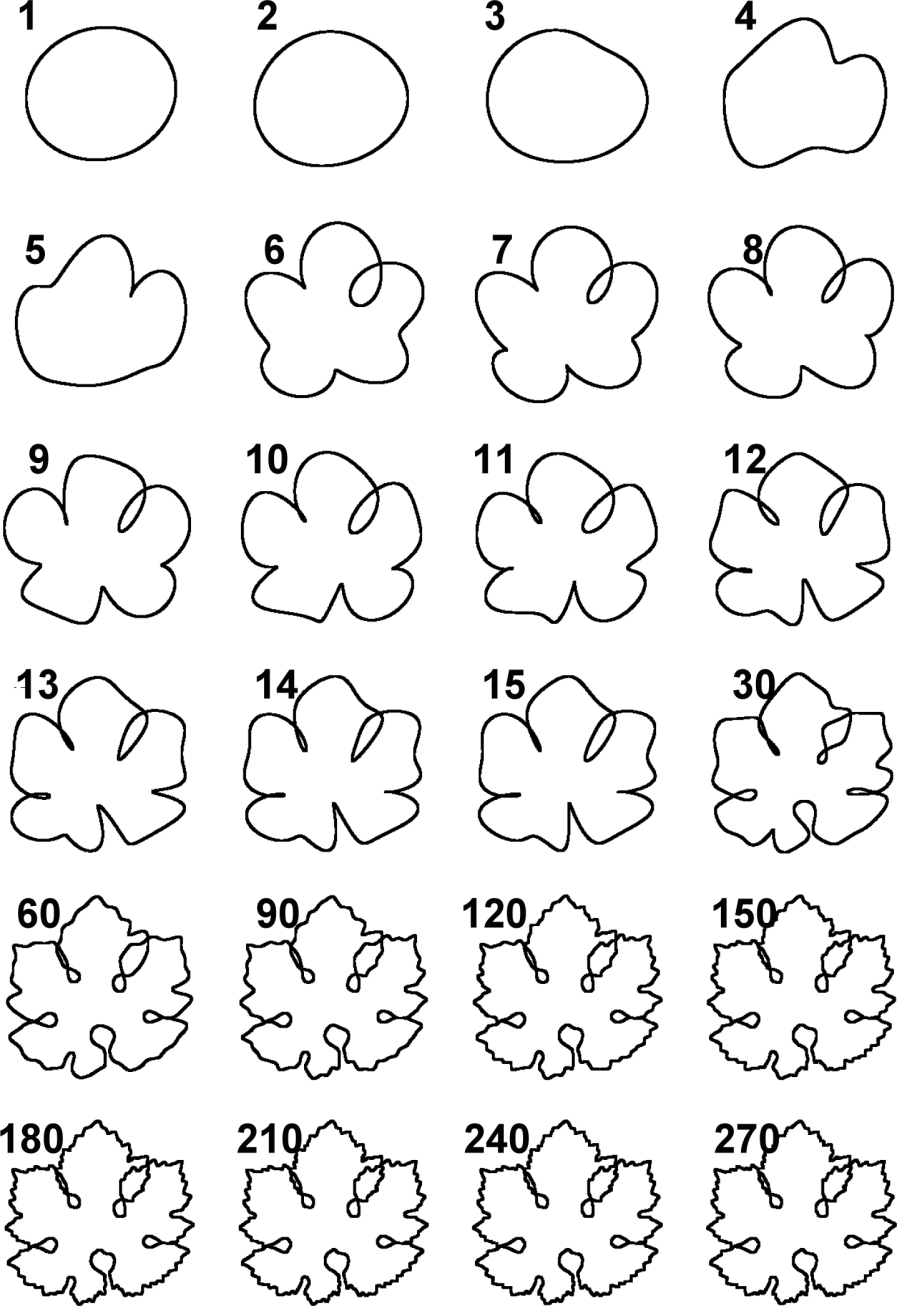
Stepwise inverse EFA transform of a single leaf contour. The image shows the leaf shape re-synthesized using the first 270 harmonics (harmonics 1 to 15, following unit steps; harmonics 60 to 270, following steps of 30). OL begin to appear from the 6^th^ and the 12^th^ harmonic.

To evaluate the effect of considering or not considering TL or OL, Fig 6 shows a collection of leaves of six grapevine cultivars (A-F) exhibiting TL and OL, and six cultivars (G-L) not exhibiting TL or OL. Cultivars A-F were processed in two ways: one using the dual threshold which recognizes TL and OL (Fig 6, + columns), and one using the default threshold method that does not recognize OL or TL (Fig 6,—columns). For a statistical comparison, the 90 contours of the three groups of contours (OL/TL+, OL/TL- and noOL/TL) where processed by elliptic Fourier analysis (EFA) [10] and the first 20 elliptic harmonics [3,11] were set as input variables for a principal component analysis (PCA), a classical multivariate exploratory method. The PCA plot (Fig 7) clearly separates the OL/TL+ group from the other two groups, indicating that TL and OL are essential shape components for discriminating the two groups of cultivars (A-F and G-L). The contribution of elliptic harmonics to generate the leaf shape, including TL and OL, can be visualized by the stepwise inverse transform of elliptic Fourier coefficients (Fig 8).

The method so far described can also be used to capture the shape of overlapping leaves. This is certainly irrelevant working with fresh samples but may be of interest with herbarium specimens whose leaves are often overlapped but can not be handled and re-positioned, because of their rigidity and fragility. However, it should be noted that this application presents two problems. One may be the contextual presence in herbarium specimens of voluminous flowers and fruits prevent the use of even a soft compression to flatten the leaves on the scan bed. A second problem is relative opacity of the paper supporting the specimens that hampers the transillumination. However, about this point I found that paper sheets weighing 120 g/m^2^ or less allow sufficiently good images to be captured using a commercial scanner with manually enhanced brightness.

**Fig 9:**
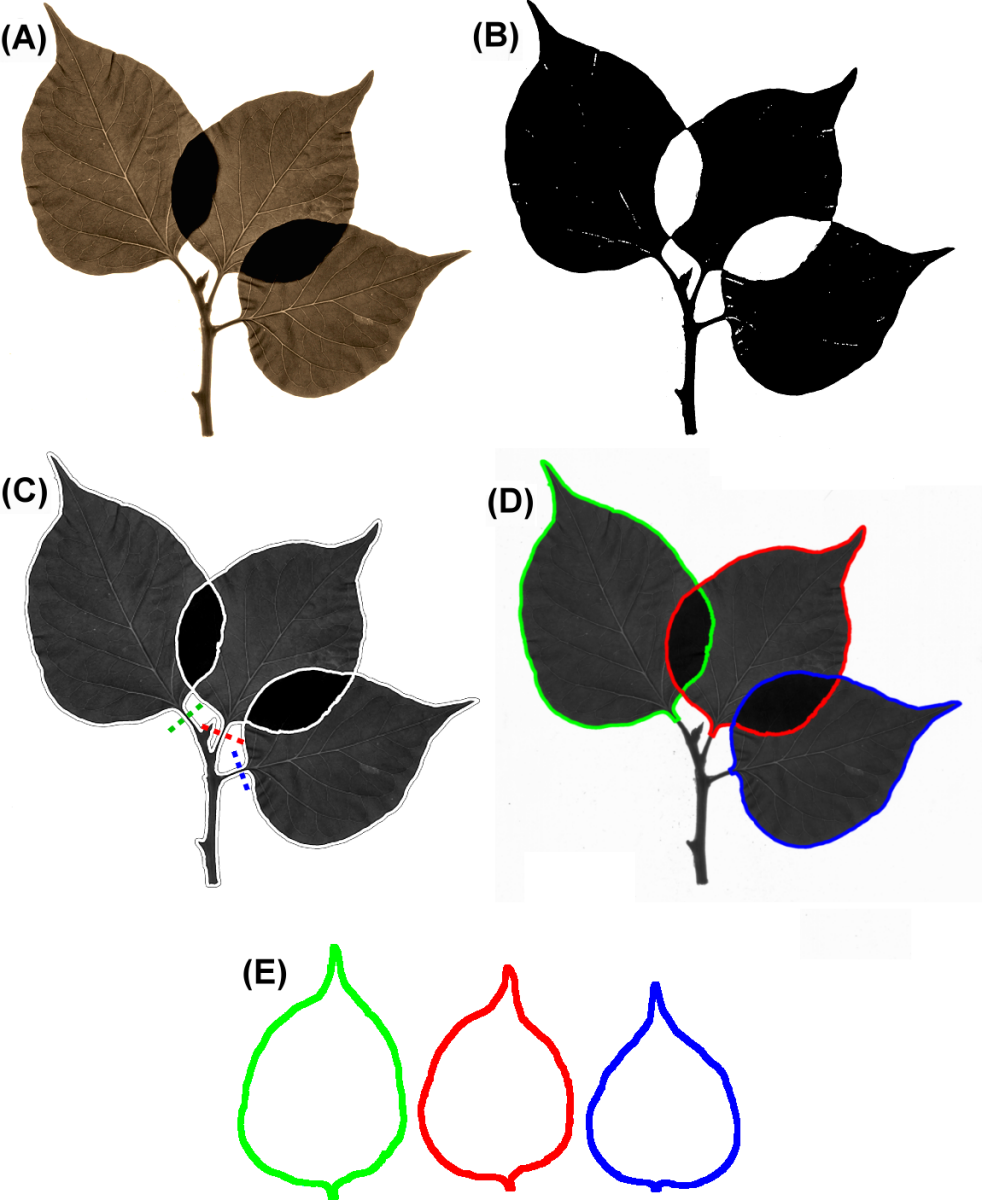
Separation of leaves of herbarium specimens. (A) Original herbarium specimen. (B) Dual threshold mask. (C) Whole specimen contour. The dashed lines indicate the cutting points. (D) Single leaf contours obtained with the cut function and traced with different colors. (E) Separated leaf contours.

**Fig 10:**
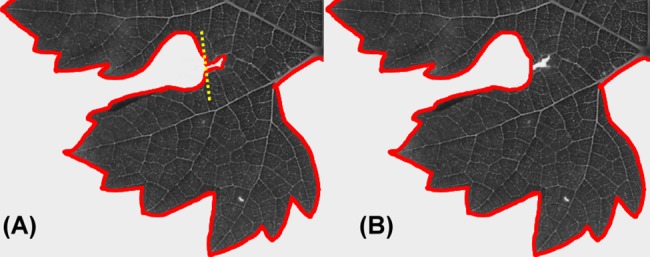
Cut utility. Contours can be splitted using the cut function to remove the petiole from the leaf, to 'repair' small breaks of the leaf border and also to separate overapping leaves attached to the same twig, as shown in Fig 9. (A) Small break of the leaf border. The yellow dashed line indicates the split line. (B) The resulting 'repaired' contour.

**Fig 11:**
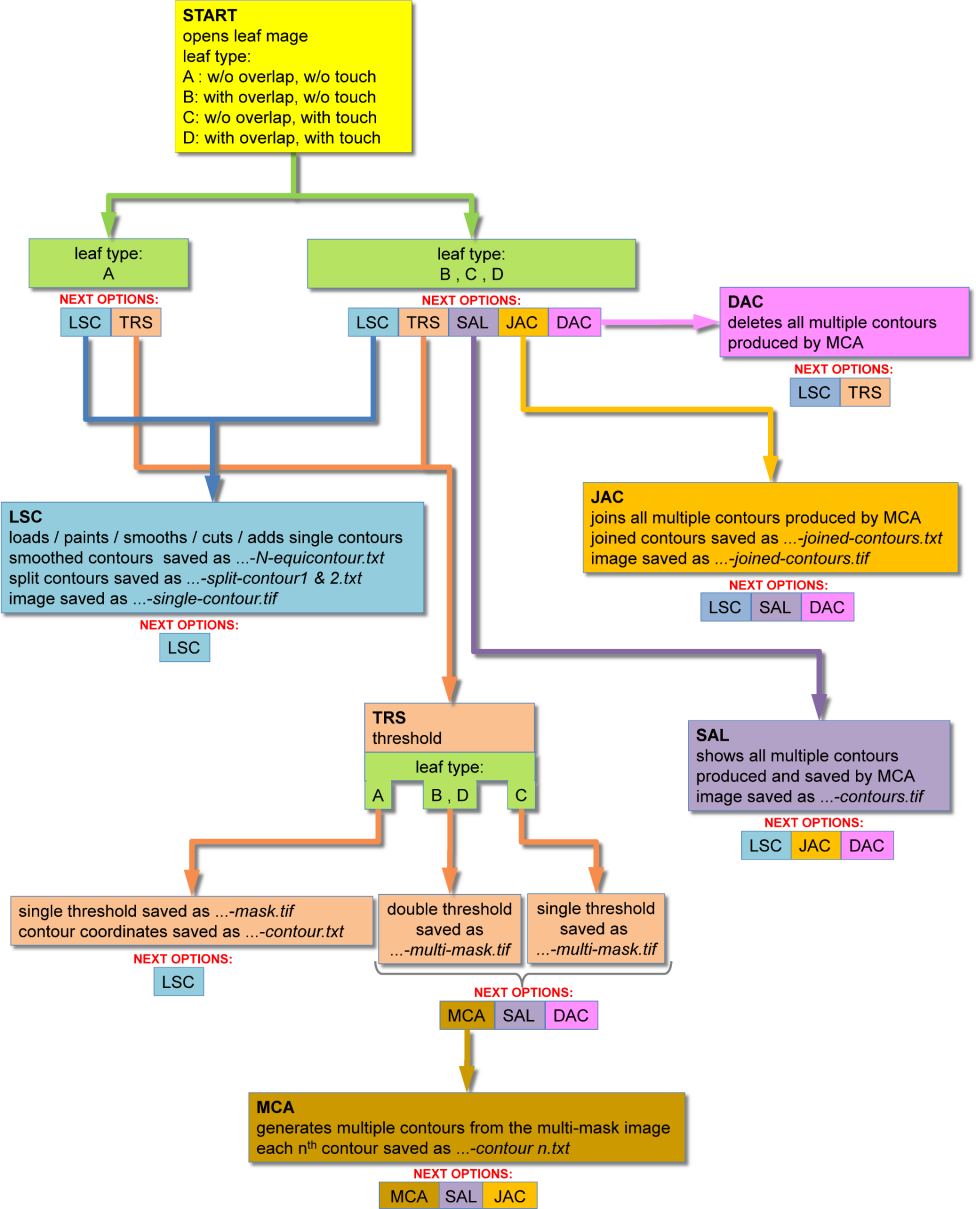
Block diagram of the ImageJ macro. The diagram shows the main functions (shown as large blocks, identified by three-letters acronims in bold fonts) and the main sequence of operations (arrows). The complete list of operations that can be executed after the completion of each function is indicated by the 'NEXT OPTIONS' acronyms, below each block. Re-start or exit from the macro can be done at any time by menu command or by the ESC-key (not shown).

Other utilities present in the macro allow the contours to be re-traced, overimposed and saved as bitmap images or coordinate files. Coordinates are saved in the raw format, the same adopted by ImageJ, so they can be directly imported and imaged by ImageJ by using the menu commands: File > Import > XY Coordinates. Contours can also be splitted. This makes it possible to cut off petioles, to separate overlapping leaves attached to the same twig (Fig 9), as well as to patch up small breaks of the leaf border (Fig 10). The whole block diagram of the macro is shown in Fig 11. The ImageJ/Fiji macro for the acquisition of complex leaf shapes is available from the S1 File. The macro performing the elliptic Fourier analysis [10] to extract the harmonic components of leaf shapes [3,11,12] is available from the S2 File.

## Supporting information

S1 File Complex leaf contours—v35. ImageJ macro code performing the acquisition of complex leaf shapes.
(TXT)

S2 File Ellitic fourier analysis—v24. ImageJ macro code performing Elliptic Fourier analysis.
(TXT)
